# Canadian Health Personnel Attitudes Toward Refugee Claimants’ Entitlement to Health Care

**DOI:** 10.1007/s12134-021-00892-4

**Published:** 2021-09-10

**Authors:** Cécile Rousseau, Joanna Anneke Rummens, Rochelle L. Frounfelker, Monica Ruiz Casares Yebenes, Janet Cleveland

**Affiliations:** 1 Department of Psychiatry, McGill University, CIUSSS Centre-Ouest de L’Ile-de-Montréal, QC H3N 1Y9 Montreal, Canada; 2 Nursing Department, Ryerson University, Toronto, ON Canada; 3 Sherpa Research Centre, CIUSSS Centre-Ouest de L’Ile-de-Montréal, Montreal, QC Canada

**Keywords:** Refugee claimants, Health care access, Canada, Health care personnel

## Abstract

Health care personnel attitudes toward refugee claimant entitlement to health care are influenced by multilevel factors including institutional and societal culture. Although individual attitudes may be modified through training, macro- and meso-issues require system-level interventions. This paper analyzes the role of individual-, institutional-, and city-level factors in shaping attitudes toward refugee claimants’ access to health care among Canadian health care personnel. A total of 4207 health care personnel in 16 institutions located in Montreal and Toronto completed an online survey on attitudes regarding health care access for refugee claimants. We used multilevel logistic regression analysis to identify individual-, institutional-, and city-level predictors of endorsing access to care. Participants who had prior contact with refugee claimants had greater odds of endorsing access to care than those who did not (OR 1.13; 95% CI 1.05, 1.21). Attitudes varied with occupation: social workers had the highest probability of endorsing equal access to health care (.83; 95% CI .77, .89) followed by physicians (.77; 95% CI .71, .82). An estimated 7.97% of the individual variation in endorsement of equal access to health care was attributable to differences between institutions, but this association was no longer statistically significant after adjusting for city residence. Results indicate that the contexts in which health care professionals live and work are important when understanding opinions on access to health care for vulnerable populations. They suggest that institutional interventions promoting a collective mission to care for vulnerable populations may improve access to health care for precarious status migrants.

## Introduction

The response in reception countries to the growing number of refugees is increasingly polarized between inclusive policies, designed to promote refugee integration and well-being, and dissuasive policies, including reduced access to services (European Union Agency for Fundamental Rights, 2017; Haas et al., 2016; Stubbe Østergaard et al., 2017). Since 1957, through the Interim Federal Health Program (IFHP), Canada has traditionally provided all refugees and refugee claimants with coverage of medical services, medications, and supplemental benefits (e.g., dentistry, eyeglasses) similar to that afforded social assistance beneficiaries (Elgersma, 2008). In 2012, the federal government made major cuts to the IFHP, ceasing almost all coverage of medication and supplemental benefits, as well as eliminating coverage of nearly all medical services for some refugee claimants (Citizenship & Immigration Canada, 2012; Ruiz-Casares et al., 2016).

The IFHP cuts were part of broader policy changes by the Conservative government that included increasing immigration detention powers and restricting access to refugee status. Jason Kenney, then Minister of Immigration, stated “This legislation will help stop foreign criminals, human smugglers and those with unfounded refugee claims from abusing Canada’s generous immigration system and receiving taxpayer-funded health and social benefits.” Conservative party messaging asserted that those targeted by the cuts were “illegal migrants” making “fraudulent claims” and “taking advantage” of Canada (Beatson, 2016; Villegas et al., 2019).

Quebec immediately implemented a program compensating for the federal cuts, and Ontario followed 18 months later (Ontario Ministry of Health & Long-term Care, 2013). Health professional organizations, including the Canadian Medical Association, opposed the changes on clinical, public health, and humanitarian grounds (Canadian Medical Association et al., 2012). Led by the Canadian Doctors for Refugee Care, a coalition of health and legal professionals waged a vigorous campaign against the cuts (Eggertson, 2013). This culminated in a Federal Court decision declaring that they constituted “cruel and unusual treatment” that “shocks the conscience and outrages our standards of decency” (Canadian Doctors for Refugee Care et al. v Canada (Attorney General), 2014). After winning the federal elections in 2015, the Liberal party reinstated full IFHP coverage in April 2016 (Immigration Refugees & Citizenship Canada, 2016).

Unfortunately, refugee claimants still have difficulty accessing health care, as numerous professionals and clinics do not offer services to this population (Cleveland & Hanley, 2018). It is crucial to understand factors, outside of public policies that dictate the legal scope of health coverage, which may influence access to care. These factors operate on the micro-level, such as attitudes of health care professionals and individual-level interactions between professionals and refugee claimants. Evidence suggests that perceived negative attitudes and discrimination of providers toward refugee claimants can have a direct impact on their willingness to access and utilize services when needed (Chase et al., 2017; van der Boor et al., 2020). In addition, meso-level, institutional factors can also influence care. The culture, policies, and procedures of individual health care institutions can impede or promote the provision of services to refugee claimants (Hudelson et al., 2014; Robert & David, 2019; Timlin et al., 2020).

There is existing research that these micro- and meso-level factors are relevant to refugee claimant access to health care in the Canadian context. For instance, a study conducted before the IFHP cuts examined the attitudes of health personnel in hospitals and primary care centers in Montreal regarding access to health care for children and pregnant women with precarious immigration status, including refugee claimants (Ruiz-Casares et al., 2013; Vanthuyne et al., 2013). This study found that stronger support for access to services was associated with being a clinician, working in primary care centers, and first-generation status. The more favorable attitudes in primary care centers may stem in part from greater proximity to refugee families, who tend to seek care at neighborhood clinics. It may also reflect differences in institutional mandates. Montreal primary care centers have historically had a populational mandate, including services for communities facing social adversity, whereas hospitals more typically provide services only to patients who either have health insurance or are able to pay, except for emergency care for life-threatening conditions Because of the potential implications of those findings in terms of policy, we decided to investigate whether the same institutional effect was present in other settings in Canada. We hypothesized that similar institutional, occupational, and personal factors might influence attitudes toward refugee claimant access to health care in both Toronto and Montreal following the IFHP cuts.

In addition, there are indications that attitudes might differ according to city of residence in Canada. Toronto prides itself on being the “most diverse city in the world” and strongly endorses multiculturalism (https://meetingoftheminds.org/opportunity-dignity-lessons-multiculturalism-toronto-25217). Diversity is considered an integral part of the city’s identity, as evidenced by its official motto, “Diversity Our Strength.” In Montreal, on the other hand, attitudes toward immigration are more ambiguous, primarily due to concerns among the French-speaking population that non-francophone immigration might jeopardize the survival of Québec’s culture (Turgeon & Bilodeau, 2014). Debates around a proposed Quebec Charter of Values polarized public debate around integration of immigrants and racialized minorities (Hassan et al., 2019). Although systemic racism has been documented in both Toronto (http://www.ohrc.on.ca/en/disparate-impact-second-interim-report-inquiry-racial-profiling-and-racial-discrimination-black) and Montreal (https://ocpm.qc.ca/sites/ocpm.qc.ca/files/pdf/P99/resume-reds_francais.pdf), our expectation was that attitudes toward refugee claimants would be globally more favorable in Toronto.

This paper on professional perceptions analyzes the role of individual- (age, gender, generation, occupation, previous contact with refugee claimants), institutional- (hospitals and Community Health Centers (CHC)), and city-level factors in shaping attitudes toward refugee claimants’ access to health care among personnel in Canadian health care institutions. We address the following questions:

1. Is there a relationship between health personnel occupation (clinical or administrative) and city of residence and attitudes toward entitlement of refugee claimants to health care? We hypothesize that clinical health personnel and individuals residing in Toronto will be more likely to endorse refugee claimant entitlement to health care than non-clinical personnel and individuals residing in Montreal.
2. Do between-institution differences account for variation in attitudes toward entitlement of refugee claimants to health care? We hypothesize that institutional-level differences will be associated with the outcome above and beyond individual-level characteristics.

## Methods

### Participants

Participants included both clinical and non-clinical staff in health care institutions in Montreal (Quebec) and Toronto (Ontario). In Montreal, participants came from 5 separate hospitals and 2 primary care centers, counted as a single institution. In Toronto, participants included staff at 9 hospitals and a network of 19 Community Health Centres (CHCs), with the latter counted as a single institution. The rationale for merging data from the primary care centers in Montreal and CHCs in Toronto was because these centers operate under a shared, distinct mission to provide services to multiethnic, marginalized communities including refugee claimants (see for example http://www.wellesleyinstitute.com/publications/community-centres-of-greater-toronto-health-equity-plan/). Thus, while personnel at these centers are not housed at a distinct institution such as a hospital, they represent networks of care with shared characteristics compatible with that of a single organization. The total number of completed survey respondents from these 16 institutions was 4207 after removal of observations with missing data on the outcome and predictor variables (*n* = 130, 2.99% of the sample).

### Data Collection

Data were collected via an online questionnaire. The survey was designed and pretested by a multidisciplinary team specializing in refugee health and policy. It was written in English and forward translated into French, with both English and French versions accessible to respondents in Montreal. The survey contained 19 multiple-choice questions, including several on attitudes regarding health care access for refugee claimants. In Montreal, the questionnaire was administered using LimeSurvey over a period of 6 weeks in May–June 2014. A link to the survey was posted on the institutions’ intranet and/or distributed through internal institutional email lists, with individual reminders sent twice during the survey period. In Toronto, the survey was administered using REDCap software over a period of 6 weeks in July–August 2015. The survey was disseminated through internal email lists, either for each occupational category or general institutional lists and/or intranet posting.

### Measures

#### Sociodemographic Characteristics

Participants self-reported all sociodemographic information. Gender was measured as a three-category variable (male/female/other); male was used as the reference group. Occupation was coded as a series of dummy variables for the following options: physicians, nurses, social workers, other health professionals, managers, administrative staff, and other. “Other health professionals” included health care service providers such as midwives, pharmacists, physiotherapists, and psychologists. The occupation category of “other” included health technicians and technologists, researchers, and trade, auxiliary, and technical service employees (maintenance, food services, trades, etc.). Physicians were treated as the reference group in the analysis.

Age was measured as a categorical variable and coded into dummy variables in the following categories: aged 29 or under, 30–39, 40–49, 50–59, and aged 60 and over. The youngest category was the reference group. Gender was measured as a three-category variable (male/female/other); male was used as the reference group (i.e., the category of the variable that serves as the basis for statistical comparison). As with other sociodemographic characteristics, generation status was coded as a series of dummy variables. Participants were asked if they were born in Canada, as well as if their mother and father were born in Canada. Individuals born outside of Canada were classified as first generation; if born in Canada with one or both parents born outside of Canada, they were classified as second generation; all others were classified as third generation. Participants who were first-generation Canadians were the reference group.

#### Contact with Refugee Claimants

Participants self-reported if they worked directly with refugee claimants (yes/no), with “no” treated as the reference group.

#### City of Residence

We included the contextual variable of city of residence (Toronto or Montreal), with Montreal coded as the reference group.

#### Access to Health Care for Refugee Claimants

Endorsement of the belief that refugee claimants should have the same access to health care as provincial health insurance cardholders was determined by yes/no response to the following question “I am in favor of providing the following group with the same health services as [provincial health insurance] cardholders free of charge: All refugee claimants living in province from the time that they apply for refugee status to the time their refugee claim is either accepted, or it is rejected and they are deported.” Responses were coded either yes or no.

### Statistical Analysis

First, we used univariate statistics to describe the sample and prevalence of endorsing refugee claimant access to health care among all participants and subpopulations based on sociodemographic characteristics. Next, we used multilevel statistical techniques, given we hypothesized that the processes that affect the outcome operate at multiple levels simultaneously (Subramanian et al., 2003). We conducted a multilevel regression analysis with 4207 people at level 1, nested within 16 institutions at level 2. Given the outcome variable is binary (yes/no), we used a multilevel logistic regression model (Austin & Merlo, 2017) fitted using the Markov Chain Monte Carlo (MCMC) estimation methods (Browne, 2017) implemented in the MLwiN program version 2.36 (Rasbah et al., 2009; Rasbash et al., 2015). All models were estimated using the logit function, and for ease of interpretation we report result in proportions, odds ratios (ORs), or both.

We developed four models guided by the approach recommended by Raudenbush and Bryk (2002), using the following sequence. We first fit a two-level null (empty) model of individuals (level 1) nested within health care facilities (level 2) with no predictor variables in the fixed part of the model. This model provides a baseline for comparing the size of the contextual variations in endorsement of equal access for health care in subsequent models. Next, we fit models with individual-level predictors. First, occupation was included as a fixed effect; second, age, prior contact with refugees, generation, and gender were added to the model. A third and final model included the contextual fixed effect of city residence as well as all other individual-level characteristics. The variation in endorsement of access to health care between institutions was estimated for each of these models.

### Ethical Issues

The Research Ethics Boards of all participating institutions approved the study. Participation was informed, voluntary, and anonymous.

## Results

Over half of study participants (59.23%) worked in Toronto health facilities, with nurses (*n* = 899) and administrative staff (*n* = 915) comprising the largest groups of health care professionals in the overall sample. The majority of respondents were female (78.77%), reflecting the composition of this population (Zelek & Phillips, 2003). A total of 1751 (41.62%) were second-generation Canadians, followed by 33.66% first-generation Canadians (Table 1). Out of the 4207 individuals included in the study, over half (61.94%) believed that refugee claimants should have access to the same health care as provincial health insurance cardholders.

**Table 1:** Sociodemographic characteristics and support of equal access to health care for refugee claimants and refugees (*N* = 4207)

Variable	Total sample	Support equal access (*N* = 2606)	Do not support equal access (*N* = 1601)
	*N*	*n (%)*	*n (%)*
Gender			
Male	883	574 (65.01)	309 (34.99)
Female	3314	2024 (61.07)	1290 (38.93)
Other	10	8 (80.00)	2 (20.00)
Age			
≤ 29	633	435 (68.72)	198 (31.28)
30–39	1152	749 (65.02)	403 (34.98)
40–49	1026	607 (59.16)	419 (40.84)
50–59	1037	584 (56.32)	453 (43.68)
60 +	359	231 (64.35)	128 (35.65)
Occupation			
Physician	577	444 (76.95)	133 (23.05)
Nurse	899	527 (58.62)	372 (41.38)
Social worker	219	187 (85.39)	32 (14.61)
Other health professionals	474	307 (64.77)	167 (35.23)
Manager	378	218 (57.67)	160 (42.33)
Administrative	915	488 (53.33)	427 (46.67)
Other	745	435 (58.39)	310 (41.61)
Institution			
0	230	207 (90.00)	23 (10.00)
1	550	357 (64.91)	193 (35.09)
2	318	232 (72.96)	86 (27.04)
3	320	184 (57.50)	136 (42.50)
4	191	129 (67.54)	62 (32.46)
5	115	74 (64.35)	41 (35.65)
6	108	67 (62.04)	41 (37.96)
7	250	166 (66.40)	84 (33.60)
8	233	131 (56.22)	102 (43.78)
12	177	91 (51.41)	86 (48.59)
13	281	168 (59.79)	113 (40.21)
14	284	150 (52.82)	134 (47.18)
15	342	201 (58.77)	141 (41.23)
16	165	88 (53.33)	77 (46.67)
17	246	109 (44.31)	137 (55.69)
18	397	252 (64.48)	145 (36.52)
City			
Montreal	1715	968 (56.44)	747 (43.56)
Toronto	2492	1638 (65.73)	854 (38.06)
Generation			
1	1416	904 (63.84)	512 (36.16)
2	1050	689 (65.62)	361 (34.38)
3	1751	101 (58.18)	728 (41.82)
Prior contact w/refugees			
No	2249	1307 (58.11)	942 (41.89)
Yes	1958	1299 (66.34)	659 (33.66)

### Individual-Level Effects

**Fig. 1:**
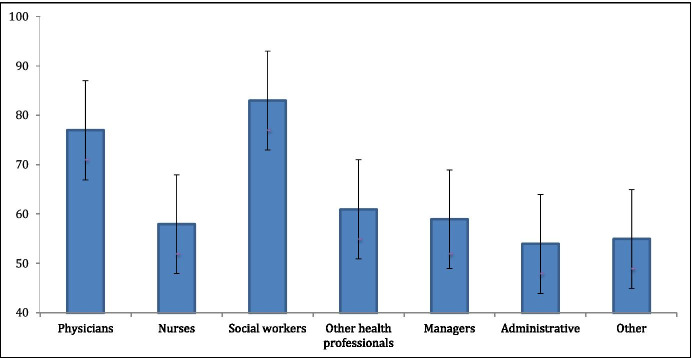
Mean probability of endorsing equal access to health care by occupation, controlling for individual and institutional effects

Table 2 contains results of the multivariate models in the order in which they were developed. The main effect for each of the individual-level covariates was estimated after controlling for all others (multivariate model 3). In terms of occupation, social workers had the highest probability of endorsing equal access to health care (0.83; 95% CI 0.77, 0.89) followed by physicians (0.77; 95% CI 0.71, 0.82) (see Fig. 1). All other occupational groups had significantly lower odds of endorsing equal access to health care compared to the reference group (physicians). There was a significant association between age and the outcome, with individuals under age 30 more likely to endorse equal access to health care for refugees than all other age groups. Likewise, those participants who had prior contact with refugees had greater odds of endorsing the outcome than those who did not (OR 1.13; 95% CI 1.05, 1.21).

**Table 2:** Association between individual- and institutional-level characteristics and endorsement of equal access to health care for refugee claimants (*N* = 4207)

Fixed parameters	Null model	Multivariate 1	Multivariate 2	Multivariate 3
*Individual predictors*		*OR (95% CI)*	*OR (95% CI)*	*OR (95% CI)*
Occupation				
Physician		1 (ref)	1 (ref)	1 (ref)
Nurse		.40 (.35, .45)	.42 (.37, .48)	.41 (.36, .47)
Social worker		1.57 (1.26, 1.97)	1.58 (1.26, 1.99)	1.56 (1.24, 1.95)
Other health professionals		.47 (.40, .54)	.48 (.41, .56)	.47 (.40, .55)
Manager		.39(.33, .45)	.43 (.37, .51)	.43 (.37, .50)
Administrative		.33 (.29, .37)	.35 (.30, .39)	.34 (.30, .39)
Other		.36 (.32, .41)	.37 (.32, .43)	.36 (.31, .42)
Age				
≤ 29			1 (ref)	1 (ref)
30–39			.77 (.69, .87)	.77 (.69, .86)
40–49			.65 (.58, .72)	.65 (.58, .73)
50–59			.60 (.54, .68)	.60 (.54, .68)
60 +			.67 (.58, .78)	.67 (.58, .78)
Prior contact w/refugees				
No			1 (ref)	1 (ref)
Yes			1.13 (1.05, 1.21)	1.13 (1.05, 1.21)
Generation				
1			1 (ref)	1 (ref)
2			1.06 (.97, 1.16)	1.06 (.97, 1.16)
3			.87 (.80, .94)	.88 (.81, .95)
Gender				
Male			1 (ref)	1 (ref)
Female			.94 (.86, 1.02)	.94 (.86, 1.03)
Other			2.83 (1.17, 6.83)	2.89 (1.18, 7.08)
*Contextual predictor*				
City				
Montreal				1 (ref)
Toronto				1.65 (1.32, 2.14)
	*Variance (SE)*	*Variance (SE)*	*Variance (SE)*	*Variance (SE)*
Random parameters				
*Level 2: between institutions (n* = *16)*	.285 (.135)	.286 (.138)	.252 (.122)	.196 (.102)

### Contextual-Level Effects

**Fig. 2:**
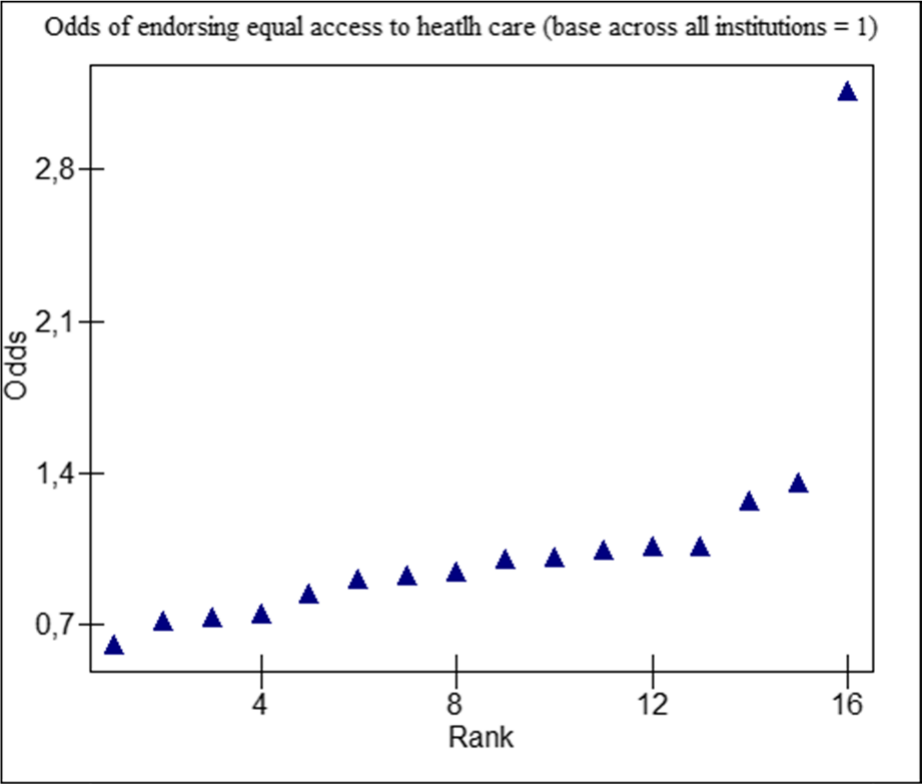
Ranking of institutional odds of endorsing equal access to health care

Ranking of the institutional odds of endorsing equal access to health care among all 16 institutions can be found in Fig. 2. Compared to a base odds ratio of 1, institutional odds ratios (ORs) ranged from 0.61 to 3.15. The outlier position of one of the institutions, the CHC network, is remarkable, with 90% of respondents endorsing entitlement to health care for refugee claimants. The null model with no predictors (Table 2, null model) indicates that an estimated 7.97% of the individual variation in endorsement of equal access to health care is attributable to systematic differences between health care institutions (${\sigma }_{u0}^{2}$ = 0.285). Between health care institutional variations diminished slightly after adding in individual-level characteristics of age, gender, prior contact with refugees, and generation (multivariate model 2). After including city residence as a contextual-level fixed effect (multivariate model 3), health care institution explained only 5.62% of the variation in the outcome and was no longer statistically significant. Individuals residing in Toronto were 1.65 times more likely (95% CI 1.32, 2.14) to approve of refugee claimant access to health care compared to those located in Montreal.

## Discussion

We hypothesized that clinicians would have higher levels of support for entitlement to health care than non-clinicians. This hypothesis was partially supported, with doctors and social workers being more favorable to access to care for claimants than administrators. Our hypotheses regarding contextual- and structural-level associations with attitude of health personnel were partially confirmed. As predicted, residing in Toronto was associated with more positive attitudes to refugee claimant entitlement to health care; institutional-level differences accounted for variation in the outcome above and beyond individual-level characteristics, but were no longer statistically significant once city of residence was accounted for.

Potential explanations for less support of entitlement among nurses and other clinical personnel as compared to physicians and social workers are amount of contact with refugee claimants, compassion fatigue, and possibly a more precarious socio-economic position. These factors may fragilize the nurses and limit their capacity to perceive and be empathic to the asylum claimants’ needs. Research indicates that favorable attitudes and beliefs about patients are influenced by level of exposure to patient experiences and predicaments (Hyman, 2009; Peláez et al., 2017). In our sample, nurses and other clinicians reported significantly less contacts with refugee claimants than physicians and social workers. In addition, in the case of nurses, literature indicates that the burden of their tasks is associated with compassion fatigue (Duarte et al., 2016; Jakimowicz et al., 2017; Roney & Acri, 2018).

Institutional effects on the outcome can be explained by examining organizational-level influences on individual attitudes and beliefs. The institutional effect is particularly striking in Toronto CHCs, where 90% of respondents supported health care for refugee claimants. Their attitude is consistent with CHCs’ mission of providing services to underserved and marginalized populations and advancing health equity (Association of Ontario Health Centres, n.d.). This suggests that a clear institutional mission may in some cases supersede all other individual and structural determinants. Literature on migrant-friendly hospitals, a European movement (Bischoff et al., 2009), indicates that a strong positive leadership can shift staff attitudes through measures encouraging cultural competence (Hudelson et al., 2014), but may be also through shared feelings of pride around the commitment to a common mission to serve marginalized communities.

Inter-city differences may be due to a number of factors. In the health sector, the Toronto Central LHIN strongly emphasizes services for newcomers (Toronto Central Health Line.ca, n.d.; Toronto Central Local Integration Network, 2016–2017). More generally, in opinion polls, Ontario residents express more favorable attitudes to “racial minorities” than Quebec residents (Wilkins-Laflamme, 2018). A 2014 poll found however that Quebecers were more favorable than Ontarians to giving refugee claimants the same health care as Canadian citizens (Forum Research), indicating that attitudes toward entitlement may vary with time and with local social benefit cultures. While a significant portion of the population in both Toronto and Montreal are reluctant to provide social rights such as public health insurance to precarious status migrants, this may be heightened in Montreal due to the more general concerns about immigration among Quebec’s French-speaking population linked to their minority national status (Banting & Soroka, 2012).

Overall, our results provide evidence that the contexts in which health care professionals live and work are important when understanding opinions on access to health care for vulnerable populations. Only after the addition of the contextual-level factor of city did between-institution variation become insignificant. The range of institutions an individual has the option of working in is clearly dependent upon the larger geographical location (in this case city) in which the person lives; these locations in turn have characteristics that may have an impact on attitudes and beliefs about access to health care. Thus, care should be taken when interpreting the contextual-level effect of city on the outcome. More specifically, city as a group-level exposure variable should be understood as a proxy for other unmeasured individual, institutional, and community factors that are associated with endorsing access to health care (Diez-Roux, 1998; Roux, 2002).

There are limitations to this study. Most importantly, this study uses cross-sectional data, so we are unable to make causal inferences on the relationship between independent variables and the outcome. Data come from a convenience sample of health care personnel who voluntarily responded to an online survey at institutions who agreed to participate in the study. Results may not be generalizable to a broader population of health care providers in more geographically diverse locations, and findings should be interpreted with caution. Response bias could over- or underestimate the relationship between predictor variables and the outcome. For instance, if individuals who completed the survey were more likely than those who did not to support equal access to health care, then we might be underestimating the relationship between sociodemographic characteristics and the outcome. Additionally, as noted in the “Methods” section, networks of community health centers in both Montreal and Toronto were treated in the analysis as a single institution. It is possible that this decision may mask between-center differences within these health networks and overestimate homogeneity of institutional culture and policies. It is to be noted that actual access to health care does not necessarily correlates with the perceptions of health care providers. Without data about actual access rates, the health care provider perceptions cannot on their own predict access to health care.

Finally, both ethical requirements to preserve institutional confidentiality and lack of data on organizational-level factors, in particular organizational culture, known to influence staff attitudes limit our interpretation of contextual effects.

### Implications and Future Research

Despite these limitations, these findings have significant implications for clinical services, policies, training, and research.

#### Implications for Health Care Policies and Practices

First the association between institution-level variation and the outcome suggests that institutional-level interventions that promote the endorsement of a collective mandate to care for vulnerable populations may improve access to health care. For example, the Migrant Friendly Hospitals Movement can be a source of inspiration to guide the leadership of Canadian health institutions (Bischoff et al., 2009). A recent analysis of approaches used by health care organizations from the USA, Australia, and Europe to be responsive to the needs of diverse populations found that organizations must make a commitment, whether it be in the form of an explicit plan or policy of good leadership, to achieve the goal of providing appropriate care for vulnerable groups (Seeleman et al., 2015). Second, in order to promote institutional change, it is essential that organizations create systems for monitoring and measuring performance related to care for marginalized populations. Collecting data on access and appropriateness of care can serve to inform quality improvement policies and strategies at the organizational level (Seeleman et al., 2015). Third, training activities aimed at informing health personnel about refugee rights in terms of health care should not only address attitudes but also take into account staff direct experience with vulnerable clients and the overall effect of institutional climate (Seeleman et al., 2015). In order to effectively address perceptions and associated emotions, experiential training models may be favored. The “GP Engagement” initiative, implemented in Melbourne, Australia, is one such promising program that targets barriers on both practitioner and institutional levels (Timlin et al., 2020). In this initiative, a Refugee Health Fellow was assigned to work with health clinics and their practitioners to understand barriers to serving refugees and engage in collaborative problem-solving (Timlin et al., 2020).

#### Implications for Future Research

(1) As noted in the limitations section, our study uses cross-sectional data. It would be important to conduct longitudinal studies that track personnel attitudes and beliefs about access to care over time, particularly to document changes that may result from the implementation of organizational policies and practices designed to target this issue. (2) The key institutional factors associated with practitioners’ attitudes and practices need to be documented. This includes organizational culture and its relation to institutional procedures, to international standards, and to equity and diversity policies. Of utmost importance is for researchers to collect data on organizational-level factors, such as policies, monitoring and evaluation practices, and practitioner training and education activities, in order to identify what institutional-level components contribute most to variations in attitudes among personnel. (3) Transnational research could also provide insights on the interaction of macro-, meso-, and micro-factors influencing perceptions about entitlement.

## Conclusion

In Europe and North America, refugee claimants and other migrants are increasingly seen negatively as “illegal” intruders likely to abuse the generosity of benevolent host countries. In Canada, the former federal government engaged in extensive public messaging about “bogus refugees” receiving “gold-plated health care” to justify its 2012 cuts to IFHP coverage for refugee claimants (Beatson, 2016; Villegas et al., 2019). This study suggests that health care providers are not immune to this discourse. The fact that over a third of study respondents opposed equal health care access for refugee claimants is all the more concerning given that refugee claimants in Canada had in fact been entitled to publicly funded health care coverage equivalent to that afforded citizens for over 50 years priors to the 2012 IFHP cuts.

In Canada, in order to preserve an equitable access to health care for these vulnerable migrants, provincial governments and health care institutions need to send a strong message to their personnel, emphasizing the fact that refugee claimants are entitled to care and that it is an important part of our mission to make sure they can access the care they need. But this is not only a Canadian problem, and health care practitioners and researchers should join forces in order to advocate for an equitable access to health care of refugees throughout the world.
